# A case report of de novo psychosis after epilepsy surgery

**DOI:** 10.1192/j.eurpsy.2022.2022

**Published:** 2022-09-01

**Authors:** C. Adão, D.F. Rodrigues, A.S. Sequeira, P. Trindade, A. Velosa

**Affiliations:** Centro Hospitalar de Lisboa Ocidental, Psychiatry Department, Lisboa, Portugal

**Keywords:** epilepsy surgery, epilepsy, Psychosis, brief psychotic disorder

## Abstract

**Introduction:**

Epilepsy is a common and severe neurologic condition, with a high prevalence of psychiatric comorbidity. Epilepsy surgery has been used for its treatment, resulting in remission or significant reduction of crisis. An improvement of previously existing psychopathology has been more frequently described, however its exacerbation or *de novo* psychopathology post-surgery has also been reported. The prevalence rate for post-surgery psychosis is 1.1%. There are no clear risk factors associated to this condition, or a proposed pathological mechanism. However, most cases described in the literature are of patients submitted to temporal lobectomy.

**Objectives:**

Description of a clinical case of a first-episode psychosis post-epilepsy surgery and review of the literature.

**Methods:**

Description of a clinical case. Non systematic review of the literature, searching the terms “psychiatric”; “psychosis”; “epilepsy surgery” in the databases Pubmed, Medline and Cochrane.

**Results:**

Male, 29-year-old patient, diagnosed with refractory temporal lobe epilepsy. Neuropsychiatric history of mixed adaptation disorder, treated with escitalopram 10 mg daily. Submitted to anterior temporal lobectomy with no complications. On the 6^th^ day post-surgery, he developed persecutory and self-referent delusions. There’s no evidence of other causal factors. Treated with paliperidone 3 mg daily with symptom remission after one week. The diagnosis of brief psychotic disorder was made.

**Conclusions:**

We present a case of a *de novo* psychotic disorder, a rare complication of epilepsy surgery. In the future, it might be interesting to study this association in detail, with the goal of deepening the knowledge of the neurobiology of psychosis, particularly the involvement of temporal circuits.

**Disclosure:**

No significant relationships.

